# Understanding critical health literacy: a concept analysis

**DOI:** 10.1186/1471-2458-13-150

**Published:** 2013-02-18

**Authors:** Susie Sykes, Jane Wills, Gillian Rowlands, Keith Popple

**Affiliations:** 1 Faculty of health and Social care, London South Bank University, 101 Borough Road, SE1 OAA, London, UK

**Keywords:** Critical health literacy, Health literacy, Empowerment, Concept analysis

## Abstract

**Background:**

Interest in and debates around health literacy have grown over the last two decades and key to the discussions has been the distinction made between basic functional health literacy, communicative/interactive health literacy and critical health literacy. Of these, critical health literacy is the least well developed and differing interpretations of its constituents and relevance exist. The aim of this study is to rigorously analyse the concept of critical health literacy in order to offer some clarity of definition upon which appropriate theory, well grounded practice and potential measurement tools can be based.

**Method:**

The study uses a theoretical and colloquial evolutionary concept analysis method to systematically identify the features associated with this concept. A unique characteristic of this method is that it practically combines an analysis of the literature with in depth interviews undertaken with practitioners and policy makers who have an interest in the field. The study also analyses how the concept is understood across the contexts of time, place, discipline and use by health professionals, policy makers and academics.

**Results:**

Findings revealed a distinct set of characteristics of advanced personal skills, health knowledge, information skills, effective interaction between service providers and users, informed decision making and empowerment including political action as key features of critical health literacy. The potential consequences of critical health literacy identified are in improving health outcomes, creating more effective use of health services and reducing inequalities in health thus demonstrating the relevance of this concept to public health and health promotion.

**Conclusions:**

While critical health literacy is shown to be a unique concept, there remain significant contextual variations in understanding particularly between academics, practitioners and policy makers. Key attributes presented as part of this concept when it was first introduced in the literature, particularly those around empowerment, social and political action and the existence of the concept at both an individual and population level, have been lost in more recent representations. This has resulted in critical health literacy becoming restricted to a higher order cognitive individual skill rather than a driver for political and social change. The paper argues that in order to retain the uniqueness and usefulness of the concept in practice efforts should be made to avoid this dilution of meaning.

## Background

Health Literacy is a term that has attracted increasing attention over the last two decades. As interest in the field of health literacy has grown, definitions have widened. Although health literacy has been argued to be a ‘*repackaging of a number of other important concepts central to the ideological commitments, and the theory and practice of health promotion’*[1] p287], the concept has generated considerable debate and achieved rapid currency in policy making [2,3]. Much of the debate has centered on delineating the concept: the domains it may include, how it is manifested and measured and whether and how the concept impacts on health outcomes and health inequalities [4-6]. Instrumental to these debates has been the contribution made by Nutbeam [7] who distinguished between basic functional health literacy, communicative interactive health literacy and critical health literacy. It is this third face of health literacy, that Nutbeam [7] presents as the cognitive and skills development outcomes which are oriented towards supporting effective social and political action as well as individual action, that this article seeks to explore.

Taken literally, the ‘critical’ aspect of critical health literacy, can be a higher level cognitive ability as suggested by McLaughlin and DeVoogd [8]. If health literacy is the ability to access, understand, appraise and apply health information [9], then critical health literacy is potentially a higher order process that could be developed through education to critically appraise information of relevance to health. This is in keeping with much of the emphasis of health literacy research which is on the skills and abilities of individuals and their participation in the creation of health. Critical health literacy is elsewhere [10] seen as empowerment where being critically health literate might mean acting individually or collectively to improve health through the political system or membership of social movements. Just as health literacy might be seen as ‘new wine into old bottles’ [11] p289] of empowerment [1,11], so critical health literacy, with its focus on community capacity to act on social and economic determinants of health, is redolent of community development. Exploring critical health literacy from this angle and borrowing from Freire [12], critical health literacy is, like community development, a process in which citizens become aware of issues, participate in critical dialogue, and become involved in decision making for health [4]. Although the 7^th^ Global Conference on Health Promotion [13] identified improving health literacy as a means for fostering community involvement and empowerment, critical health literacy may be seen as the neglected domain of health literacy, rarely achieving any focus or interventions that claim to be working towards this outcome. There are those that argue that the lack of attention given to the psychological constructs within the definition of critical health literacy results in health literacy acquiring a rather cognitive focus and that health outcomes are more likely to be achieved when the dichotomy between knowledge and psychological constructs are overcome [14]. Critical health literacy may offer the opportunity to achieve this. The lack of attention the concept has been given may be due to a lack of conceptual models and frameworks that explore critical health literacy [7,15]. Alternatively, it may be the result of difficulties and confusion in grasping what exactly empowerment based skills involve and how the concept can be taken forward [16]. While such confusion exists any potential that this concept may have to offer cannot be realised and tools to measure it accurately cannot be developed. A systematic analysis of the concept of critical health literacy that explores definitions and understandings of the term in both academic literature and as held by practitioners and policy makers may help to reveal whether it is indeed a useful and unique concept.

A search of Medline using the term ‘health literacy’ showed 4115 articles had been published since 1991. A separate Medline search using the term critical health literacy identified only 39 articles. Much of the literature on health literacy focuses on different typologies that attempt to distinguish different domains or components of health literacy [4,17,18]. Chinn’s recent [19] review and critical analysis of critical health literacy identifies three domains that make up the concept; that of critical appraisal of information, understanding social determinants of health and collective action. This is an important contribution in creating clarity of meaning and understanding. However, there is a real need to analyse the concept in a far more systematic and rigorous way.

Concepts are important in describing and explaining phenomena and examples from numerous professional fields show that they underpin and explain practice for example the concepts of faith, [20] and self-care [21] yet they may be poorly delineated. As concepts become more widely used in the literature, their use may become expanded and as a result become confused with similar concepts [22] resulting in difficulties in communicating the phenomena, in evaluating its strengths and weaknesses [23] as well as to assess its unique nature. The relationship between concepts and theory is discussed widely with theory often being described as being built from ‘conceptual bricks’ [24,25]. Part of the literature on health literacy posits that there is a causal pathway whereby low levels of health literacy contribute to ill health [5] and that health literacy is an outcome that is co-created by patients and health care professionals [6]. Such theories are built on a concept of health literacy and yet it is acknowledged that confusion exists around the concept [26] therefore providing a poor basis for theory. Critical health literacy, while being seen as part of an emerging *‘third generation of health literacy development’*[27]p2], also displays elements of confusion and overlap with other concepts. This is then a crucial time to systematically and rigorously analyse the concept itself to enable appropriate theory, well grounded practice and accurate measurement tools to emerge.

## Methods

Concept analysis is a well established methodology that has been used to analyse many concepts key to public health and health promotion including cultural competence [28] empowerment [29], participation [30] equity [31] and critical media health literacy [32]. There are numerous methods of concept analysis adopting slightly different approaches but which always follow a systematic and staged process of identification and analysis. Common to most of the methods [23,25,33,34] is the systematic analysis of key elements of the concept such as (a) the attributes of the concept which refers to the key characteristics that define the concept, (b) references or what the concept is used to refer to, (c) antecedents or the factors that need to be in place in order for the concept to occur, (d) consequences or what happens as a result of the concept, (e) surrogate terms that could be used instead of the concept and (f) resemblant terms or other concepts that show similarity.

Concepts are abstractions that are expressed in some form and through repeated public interaction a concept becomes associated with a particular set of attributes and is thus publicly manifested though behavior and linguistics [35]. As a result, concepts are subject to continual change and definitions and characteristics may vary according to different contexts such as time, place, discipline and theoretical perspective [23] Rodgers offers the example of the concept of ‘health’ which is understood very differently according to contexts and has in some contexts of time, culture and discipline focused on the absence of disease while in other contexts alludes to more positive understandings associated with well-being [23]. As concepts are subject to continual change they do not have a strict set of attributes but rather a cluster of attributes which may be prioritised differently by different groups of people or at different times. This understanding of concepts and the importance of acknowledging and identifying the contextual variations led to the adoption of the evolutionary concept analysis method developed by Rodgers [23] with its emphasis on inductive processes and its commitment to contextualism.

**Figure 1:**
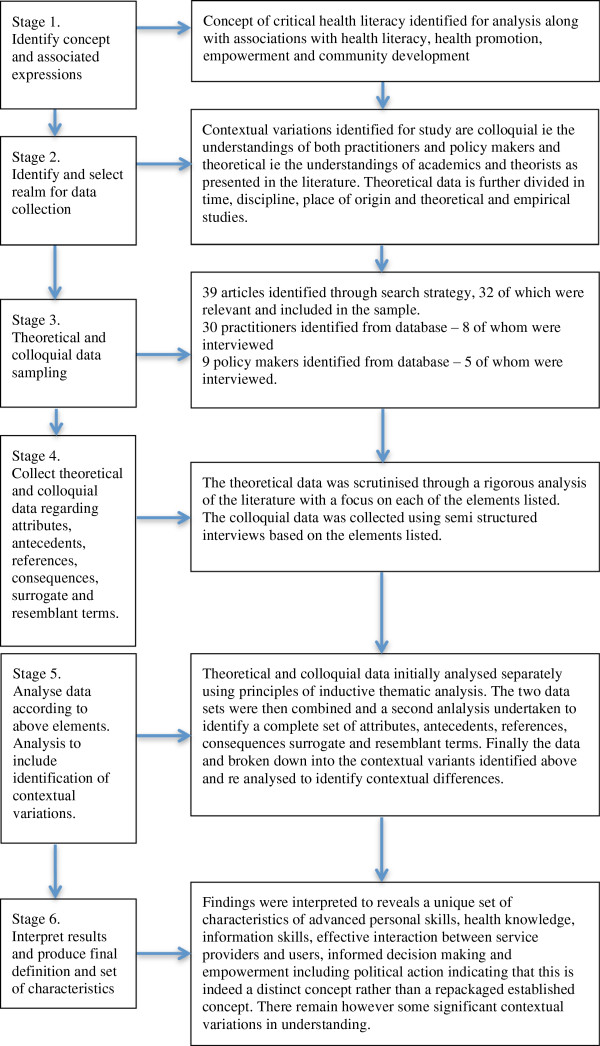
Stages of the evolutionary concept analysis process applied to critical health literacy.

This study has adapted the evolutionary concept analysis process developed by Rodgers [35] in order to incorporate the ideas of Risjord [36,37] who argues that there is a need for a further contextual distinction to be made beyond time, place and discipline. This distinction is between a ‘theoretical concept analysis’ which aims to represent concepts as they appear in a particular body of scientific and theoretical literature and a ‘colloquial concept analysis’ which aims to represent the concept as used by a particular group of people. The method adopted here acknowledges that a gap frequently exists between academic understandings of a concept and the understandings of practitioners who may be less influenced by theoretical ideas and more influenced by experience and the practicalities of delivery. An analysis that seeks to understand a concept holistically and across its contextual realms needs to explore both sets of understandings. Risjord [36,37] presents these two aspects as two distinct approaches with different aims. However, he goes on to argue that there is space for a mixed analysis and that some forms of concept analysis do require both so that the phenomena can be understood across different contextual settings. While Risjord has not applied the approach, this study has incorporated these ideas into an adaptation of the evolutionary concept analysis in order to capture both the theoretical and practice based understandings of the concept. The result is a method that has six stages represented in Figure 1 whereby the theoretical representations of critical health literacy are examined through a scrutiny of the relevant literature and the colloquial interpretations of the concept are examined through interviews with practitioners and policy makers with an interest in the field.

The sampling of the theoretical and colloquial data were undertaken concurrently. Theoretical sampling involved literature identified through databases including CINAHL, ASSIA, Medline, ERIC, Education Research Complete, Cochrane, Centre for Reviews and Dissemination, CommunityWise, IBSS, British Nursing Index and the Index to Theses. This was complemented by a search via Google and Google scholar and a citation search.

Searches were set to return any article produced, in English, since 1995 using the search term ‘critical health literacy’. From all of the searches, a total of 39 different articles were found. Of these, six were subsequently removed for lack of relevancy (if the concept was only mentioned once but not described or discussed) and one was unavailable via the British Library. A final total of 32 articles were included in the theoretical analysis.

The colloquial sample included two groups of professionals with an expressed interest in the field of health literacy; policy makers (those working at a strategic, planning or policy level either nationally or locally) and practitioners (those working in a health context directly with members of the public through the provision of projects or services). The UK Health Literacy Group (http://www.healthliteracy.org.uk/), a special interest group for the Society for Academic Primary Care, was identified as a point of access for both these groups as its membership consists primarily of practitioners and providers from health care services and non-governmental organisations, policy makers and academics with an interest in health literacy. It was important to access a special interest group because participants needed to have an awareness of the concept in order to discuss their understanding of it. A general sample of practitioners and policy makers would not have enabled this insight. Academics were screened out of the membership list as their views had already been captured through the theoretical concept analysis. Those members who had not given permission to be contacted by other members were also excluded. A total of 30 practitioners and nine policy makers meeting the inclusion criteria were identified. These were invited, by email, to participate in a telephone interview as part of the study. A total of eight practitioners and five policy makers took part in the study between April and May 2011.

The theoretical data were read initially as a familiarisation process, then reread once or twice more to identify specifically the attributes, surrogate terms, resemblant terms, references, antecedents and consequences that related to critical health literacy within the text. As the data was collected, phrases, themes and quotations relating to the above elements were recorded onto a data matrix. The colloquial data was collected through semi structured interviews carried out by telephone due to the wide geographic locations of the participants. The interviews were in depth and lasted up to 45 minutes. The interview schedule was designed carefully to represent the same areas of inquiry that were used to interrogate the theoretical data. Each interview was recorded and transcribed.

The research project complies with the ethical principles outlined in the World Medical Association Declaration of Helsinki (http://www.wma.net/en/30publications/10policies/b3/) and was submitted to and approved by London South Bank University’s Research Ethics Committee.

## Results

This section contains the key findings of the combined analysis and includes the most significant contextual variations that emerged as part of the process.

Attributes: what are the key characteristics of critical health literacy?

Key characteristics include having advanced personal and social skills including confidence, social and communication skills, self efficacy and interpersonal skills. As Wang [38] states:

*‘It implies a significant level of …personal skills and confidence.’ *[38] p 271]

It also involves the ability to access, manage, assess the credibility, understand and critically appraise information on health related issues:

*“Being able to decipher information, decode, but on top of that to have an understanding, a critical awareness of what underlies that information, so it would be a conceptual awareness.’* (participant 4)

This entails having a level of health knowledge including a level of familiarity with health terms and medical terminology, being informed about health issues and understanding these issues:

*‘It’s about whether individuals have an understanding around a wide range of issues to do with health’* (participant 21)

Another characteristic of critical health literacy is being able to contextualize information, apply it to one’s own situation, judge risk, act on information and thus share the decision making with health professionals. The focus on an ability to contextualise information was captured by Kickbush [39]:

*‘…involves understanding and ability to judge, sift and use information provided in the context of one’s own life – this is the key element of critical health literacy…’*[39] p292]

Whilst these characteristics relate to individual abilities, critical health literacy is also seen as arising from the relationship between services and individuals and an ability to interact effectively. This involves an ability to navigate services but beyond this to advocate and articulate oneself confidently when communicating with a health professional and where necessary question or challenge a professional in a constructive way as one participant demonstrated in their reflection on their own experience as a patient:

*‘I don’t just receive information, sitting there quietly absorbing it and making sense of it. What I need to do is also question, including occasionally challenging.’* (Participant 20)

This level of effective interaction is not only dependent on the skills of the individual but also on the skills of the professional who must be able to explain things clearly and provide information that is appropriate for patients. The contextual analysis showed a variation on this point between the colloquial and theoretical data. The professional participants placed an emphasis on the skills and role of the health practitioners in the creation and existence of critical health literacy. This was a theme that was only touched upon within the academic literature but which was central to the colloquial sample who stressed that critical health literacy would only exist if there was a commitment from health practitioners to provide accessible information and to engage in shared decision making.

Another characteristic of critical health literacy can be broadly described as empowerment by which a person has an understanding of the determinants and the policy context of health, an understanding of opportunities to challenge these determinants and policy and motivation and actual action at apolitical and social level. The most frequently cited reference to this point was that made by Nutbeam [7]:

*‘…the cognitive and skills development outcomes which are oriented towards supporting effective social and political action…’*[7] p 265]

This empowerment may exist at an individual level but may also demonstrate collective understanding and exist at a population or community level. As such it represents an asset rather than a deficit or lack of skills in an individual or community. The contextual analysis showed, however, quite stark variation in how this theme was understood and prioritised within the different contexts analysed. While this characteristic of empowerment was a strong and clearly articulated attribute within some of the academic articles [7,38,40,41] and by some of the colloquial sample, it was not universally emphasised. The analysis of theoretical literature shows that there has been a decrease in reference to empowerment, action at a social and political level and the conceptualisation of critical health literacy existing at a population as well as an individual level over the last five years. The contextual analysis also demonstrated that sources from a medical discipline were less likely to identify political and social action as an attribute of critical health literacy than were public health sources which focused more on cognitive critical analysis and decision making skills [42,43]. None of the empirical articles that derived from original research identified this as an attribute while theoretical articles [7,44-47] were much more likely to.

The final attribute is that of critical health literacy being a learned and movable state that may change with time or the circumstances of peoples lives:*References: what is critical health literacy used to refer to?*

*‘I think the main things are that, you know, that I feel people can maybe move up and down the levels. Depending on the kind of situation they’re in.’* (participant 14)

The concept of health literacy is most commonly used in reference to individuals and is seen as a set of skills or competencies. For some [7,48,49] it could also refer to communities or population groups as well as individuals and some (largely within the colloquial sample), also used it in reference to a relationship between individuals and professionals.

Antecedents: what needs to be in place for critical health literacy to occur?

Familiarity with health issues and services as well as an interest and motivation to find out more about health issues is a precursor of critical health literacy:

*‘even well educated people can struggle with health literacy because of the lack of familiarity and very often going very long periods without even having to engage with health services or think much about personal health.’* (participant 20)

This motivation may be triggered by personal experience of particular health issues, through social influences or through personal determination.

In order for critical health literacy to emerge an individual would have a wide skill set of literacy and language skills, critical appraisal skills, cognitive skills, personal and social skills and functional and interactive health literacy skills. While the majority of the theoretical and colloquial data argued that functional and interactive health literacy skills and actual literacy and language skills needed to be in place in order for critical health literacy to emerge, there were a minority who strongly opposed this position. For this minority, who often referred to a the Frierian approaches of critical consciousness raising [12] as indeed did Nutbeam’s original article [7], the existence of functional literacy skills of individuals was a less important area of focus:

*‘..they may be great at speaking and listening, they may be able to stand up for themselves quite well and may have an understanding of critical health literacy that isn’t dependent on their reading and writing… so those basic literacy reading and writing skills are a building block for critical health literacy but not an absolute requirement in some cases’* (participant 4)

For critical health literacy to be developed there would be formal, structured but supportive learning environments with a change in focus for health education programmes away from information giving to skills development and understanding of health inequalities and the determinants of health based on principles of community development:

*‘Within this paradigm, health education may involve the communication of information, and development of skills which investigate the political feasibility and organizational possibilities of various forms of action to address social, economic and environmental determinants of health.’*[7] p 265]

Another antecedent was political will, that is political recognition of the value of critical health literacy as well as the drive and resources coming from a political level to support the development of critical health literacy skills:

*‘But I think it needs – it does need – if there was a policy drive. If there was a condition around a policy drive to bring together people who matter, people who sign up to it.’* (Participant 39)

The contextual analysis again showed some mixed understandings and emphasis in this area. Professionals were far more likely to emphasise the theme of political will in creating critical health literacy including the need for any work to develop critical health literacy to be resourced and led at a political and strategic level in order for it to be effectively implemented. This was an area that received very little discussion in the academic literature.

The development of communication skills amongst health professionals to ensure information is presented in an understandable way and that there is a commitment to shared decision making was seen by professionals and policy makers to be important.

Consequences of critical health literacy: what happens as a result of critical health literacy?

The consequences of critical health literacy that were identified in the literature and by professionals and policy makers were supposed or anticipated rather than demonstrated through research and four themes were identified. The first theme was an increase in self efficacy including increased levels of personal involvement, action and control over health issues that affected an individual’s life, shared decision making and self management of care as captured by Ishikawa and Yano [49].

*‘…may be related to perceived control over one’s health and self-efficacy to participate in the health care process directly.’* p118

Critical health literacy would also result in improved quality of life, health behaviour and outcomes:

*‘Being able to kind of look after your health and respond to your own health issues. So, my personal point of view, obviously better health outcomes.’* (participant 14)

A critically health literate person would make more effective and efficient use of services:

*‘Critical health literacy as a compass, guiding patients successfully through complex and non transparent health markets.’ *[50] p38]

Critical health literacy was also seen as an individual and population outcome in which there would be increased levels of social capital, understanding and questioning of the determinants and inequalities of health and increased levels of social and political action and change. The contextual analysis again showed that this has been identified as a consequence less frequently in papers published in the last five years. The contextual analysis also identified that theoretical data from Public Health sources identified a far broader set of consequences, including empowerment and political action, than those in the medical literature which focused far more on improved heath related behaviour and outcomes as well as use of services.

Surrogate terms – do the characteristics mirror those of another concept?

It is possible that the characteristics of a concept may mirror those of other concepts which become known as surrogate terms. While the literature and professionals identified several surrogate terms, none emerged frequently or consistently suggesting there is no other term that captures the same characteristics of this term.

Resemblant terms – do the characteristics resemble those of other concepts?

Again, the literature and participants referred to a large number of terms that reflected some, though not all of the attributes, antecedents and consequences of critical health literacy. Those that emerged most frequently and appear to have the most in common with critical health literacy were empowerment, self-efficacy, health literacy, critical appraisal, critical consciousness and advocacy.

## Discussion

It is recognized that the sample size of the colloquial data is small and represents only a UK perspective but is likely to represent how the concept is used in discourse and policy. No other studies have been identified that seek to explore the concept from both a theoretical and professional perspective. Interviews were in depth and the highly systematic analysis of the concept of critical health literacy presents a more detailed and nuanced understanding than previous discussions. The analysis shows the concept to be a distinct concept with a unique set of attributes and antecedents. Analysis of the contextual variations of the concept (that is, the differences studied in how the concept is understood across time, geography, discipline and across the theoretical and colloquial data), however, reveals that there is not a consensus of understanding of the term and that, in particular, there are distinct differences in the way that academics and professionals interpret the term. Thus, the findings do not represent a definitive list but rather a cluster of attributes, which may be prioritised differently in different contexts, based on the principles of ‘family resemblances’ [51] These differences demonstrate the importance of a systematic clarification of the concept, such as this study provides, before work can be done to look at how the concept and theories around it might be developed, how it might be applied in practice and its existence measured.

A key finding to emerge from this study is that critical health literacy is a unique concept. Several resemblant concepts were identified, the most frequently mentioned being empowerment. Indeed Tones [1] argues that the meaning of health literacy has already been more appropriately mapped by the existing conceptualization of community and individual empowerment. There are many definitions of empowerment and several concept analyses have been undertaken [52,53] suggesting that it too is widely used to describe an outcome but its characteristics are less clear. Gibson’s [28] concept analysis of empowerment, which is frequently referenced, shows a clear overlap of attributes, antecedents and consequences with critical health literacy. However, there are key distinctions. In particular, attributes of empowerment do not include direct reference to the theme of information skills, which is so central to critical health literacy. The ability to access, understand and manage health information as well as the ability to assess its credibility and to critically analyse and where appropriate challenge the information may possibly be skills held by an ‘empowered’ individual or community but they are not essential to empowerment in the way that they are to critical health literacy.

Despite the unique nature of the concept, the study identified several contextual variations in its interpretation and use and these need to be looked at more closely. The term critical health literacy has become part of the debates on health literacy, stemming largely from an original paper by Nutbeam [7]. In his article, Nutbeam presents Freebody and Luke’s [54] classification of literacy into basic/functional literacy, communicative/interactive literacy and critical literacy. Within this framework, critical literacy is defined as ‘*more advanced cognitive skills, which together with social skills, can be applied to critically analyse information, and to use this information to exert greater control over life events and situations.’*[7] p264] He later goes on to apply this to a health context and provides a more specific and applied explanation of ‘critical health literacy’ which discusses far more explicitly the attribute of *‘skills which investigate the political feasibility and organisational possibility of various forms of action to address social, economic and environmental determinants of health’* and the links to population health as well as individual health: *‘This type of health literacy can be more obviously linked to population benefit, alongside benefits to the individual.’*[7] p265]. Subsequent citations of Nutbeam’s work, however, frequently use the definition of critical literacy as a definition of critical health literacy [42,49,55] to the point at which it has almost become the accepted definition. While this definition alludes to greater individual control over life events, its lack of specific reference to social and political action and existence at a population level means these elements are in danger of becoming lost so distorting the original meaning and emphasis.

It is not possible to be conclusive about the reasons why the interpretation of critical literacy has, in some quarters, superseded Nutbeam’s more comprehensive, applied and political definition of critical health literacy. However, the dominance of one interpretation over another cannot be overlooked as it limits the concept to a higher order cognitive individual skill rather than a driver for political and social change. In order to understand whether this enhances the concept for theoretical and practical use or whether it simply dilutes it and makes it more resemblant of other existing concepts, it is important to consider the possible reasons for this fundamental change in conceptual understanding. One possible reason is that the debates around health literacy have been dominated by the functional domain which focuses on technical, practical and individual skills sets [19]. Within this dominant discourse assuming critical health literacy to simply be a set of higher order cognitive skills may be a natural progression. Secondly, acknowledging the place of social and political action and existence at a population level within the definition of critical health literacy requires a consideration of how such skills could be developed and measured, a challenge that is perhaps more complex and which receives less attention. Indeed, a socially and politically activated community with a critical understanding of health and its determinants may be a less desirable outcome for some. Thirdly, it could also be because the paradigm in which the discourse has taken place has narrowed the interpretation. The debates around both health literacy and critical health literacy have remained largely within the health field and this has perhaps constrained interpretations. Zarcadoolas et al. [4] have pointed to the need to make use of the developing knowledge of other academic arenas and other types of literacies such as science literacy, cultural literacy and civic literacy and their relevance for making health decisions.

It is useful to look at other professional fields. There are, for example, clear overlaps between the consequences or potential outcomes of critical health literacy and the objectives of community development work and yet the search for this study revealed no citations within the community development literature, despite this apparent affinity. This illustrates the contextual influence on concepts highlighted by Rodgers [23] and raises the question of why, if critical health literacy has currency and utility, the concept is not deployed outside the health field. Is it because the concept is not useful within community development because it lacks relevancy, is it because there is too little theoretical basis to inform the complex pathways of the community development process or is it simply because the concept is too new and has yet to find an entrance into the community development discourse? Taking the concept beyond the debates of the health environment might allow the fuller, more political and community based definition of critical health literacy to be understood and developed while identifying potential routes for the development of critical health literacy.

A further difference in understanding revealed by this study is the relationship between critical health literacy and the other domains of functional and interactive/communicative health literacy. The majority of both colloquial and theoretical data argue that functional and interactive health literacy including actual literacy and language skills need to be in place in order for critical health literacy to emerge. Sections of the data however either opposed this position or placed less emphasis on the importance of technical skills arguing that critical health literacy can emerge without the existence of such technical skills. This group make comparisons to Freierian approaches of critical consciousness raising [12], as does Nutbeam’s original article [7] whereby a liberatory education is achieved through raising levels of awareness, in particular awareness of oppression, rather than through a functional curriculum. However, if the existence of information skills is key to critical health literacy and is one of the attributes that makes it a distinct and unique concept, the link with literacy skills cannot be ignored. The information skills described may equally be applied to non written sources such as discussion based or visually presented information. However in a world where the vast majority of health information remains in written form a level of fundamental literacy skills must remain an advantage.

The role of health professionals in creating critical health literacy which was referred to by professionals and policy makers, turns critical health literacy from a set of skills and competencies existing within, and owned by, an individual or community into a transactional concept. As such, it depends on the motivation and development of skills within the individual or community but also requires collaborative efforts at a structural level in order to fully prosper. The inclusion in the colloquial data of a need for there to be a political will and driver in order for critical health literacy to develop, demonstrates that when applying the academic ideas in a practice context, professionals clearly locate responsibilities beyond the individual level. This maps shifts in focus of discussions around health literacy. In 2004, for example, the Institute of Medicine Committee on Health Literacy, called for policy makers to consider the interaction between the skills of individuals and the demands of social systems and make needed correctives [9]. This development of the concept is part of a natural evolution that is in line with the evolution of the broader concept of health literacy, rather than an area of disagreement between the theoretical and colloquial data.

## Conclusion

This evolutionary concept analysis of critical health literacy has provided a thorough and systematic analysis of the definition and use of the concept of critical health literacy across several contexts including both theoretical literature and the colloquial data from practitioners and policy makers. It reveals a unique set of characteristics of advanced personal skills, health knowledge, information skills, effective interaction between service providers and users, informed decision making and empowerment including political action indicating that this is indeed a distinct concept rather than a repackaged established concept. Not only is it a distinct concept but the potential consequences identified by this study, as well as its relationship to Health Literacy, demonstrate its importance to public health and health promotion in improving health outcomes, creating more effective use of health services and reducing inequalities in health. However, the contextual analysis shows that there is not consistency in how the term is understood by academics, practitioners and policy makers and that some of the key attributes initially presented as part of this concept, particularly those around empowerment and social and political action existing at an individual and population level, are in danger of losing their importance. In order to prevent the concept losing its unique nature, this narrowing of its definition must be avoided and the concept must not be allowed to get lost in the wider health literacy debate. This might be achieved through further research and discussion to examine how critical health literacy might be developed in practice and ultimately measured. From this the link with health outcomes can more easily be explored. In order to do this, a closer engagement with the field of community development to explore the concept further within this professional context of skills, understandings and associations with empowerment and political action is recommended.

## Competing interests

All authors declared that they have no competing interest.

## Authors’ contributions

SS conceived of the study, adapted the concept analysis research model, carried out the data collection and analysis and drafted the article. JW and GR contributed to the conception and design of the concept analysis and has been involved in the direction and content of the manuscript. KP participated in the supervision of the study and advised on drafts of the paper and approved the final manuscript. All authors read and approved the final manuscript.

## Authors’ information

SS is Senior Lecturer in Public Health and Health Promotion with a particular interest and professional background in health promotion, community development and policy making. She is currently part way through a PhD exploring the concept of critical health literacy and its usefulness and relevance to public health practice.

JW is Professor of Health Promotion and has published widely on the discourse and practice of health promotion and latterly, on the utility of health literacy as a concept to explain the empowerment of individuals and communities and their abilities to access information and navigate health systems.

GR is an academic General Practitioner. She is Professor of Primary Care and Public Health in the Faculty of Health and Social Care at London South Bank University, UK. Her research focus is on Health Inequalities, in particular the impact of low Health Literacy on health. She is the UK Department of Health GP Health Literacy Champion. She set up, and now chairs, a Health Literacy group to raise awareness of HL and develop the HL evidence-base in the UK.

KP is Professor and Head of Social Work at London South Bank University. He has published extensively in the areas of community development and social work and is joint editor with Gary Craig, Marjorie Mayo, Mae Shaw and Marilyn Taylor of *The Community Development Reader* published in 2011 by Policy Press. With Paul Stepney he jointly authored the text *Social Work and the Community: a critical context for practice*, published in 2008 by Palgrave. He is a member of the editorial board of the *British Journal of Social Work*, and the *Community Development Journal.*

## Pre-publication history

The pre-publication history for this paper can be accessed here:

http://www.biomedcentral.com/1471-2458/13/150/prepub
